# Dexmedetomidine-ketamine combination versus fentanyl-midazolam for patient sedation during flexible bronchoscopy: a prospective, single-blind, randomized controlled trial

**DOI:** 10.1186/s12890-024-02988-w

**Published:** 2024-06-26

**Authors:** Frimas Apostolos, Zias Nikolaos, Martinos Charalampos, Karkoulias Kyriakos, Fouzas Sotirios, Voyagis Gregorios

**Affiliations:** 1 Athens Naval Hospital, Athens, Greece; 2 Medical School, University of Patras, Patras, Greece

**Keywords:** Dexmedetomidine, Ketamine, Flexible bronchoscopy, Sedation, Desaturation, Adverse events

## Abstract

**Background:**

Sedation during flexible bronchoscopy (FB) should maintain an adequate respiratory drive, ensure maximum comfort for the patient, and warrant that the objectives of the procedure are achieved. Nevertheless, the optimal sedation method for FB has yet to be established. This study aimed to compare the standard recommended combination of midazolam-fentanyl (MF) with that of dexmedetomidine-ketamine (DK) for patient sedation during FB.

**Methods:**

Patients subjected to FB were randomly assigned to a DK (*n* = 25) and an MF group (*n* = 25). The primary outcome was the rate of critical desaturation events (arterial oxygen saturation < 80% with nasal oxygen supply 2 L/min). Secondary outcomes included sedation depth, hemodynamic complications, adverse events, and patient and bronchoscopist satisfaction.

**Results:**

The incidence rates of critical desaturation events were similar between the two groups (DK: 12% vs. MF: 28%, *p* = 0.289). DK achieved deeper maximum sedation levels (higher Ramsay - lower Riker scale; *p* < 0.001) and was associated with longer recovery times (*p* < 0.001). Both groups had comparable rates of hemodynamic and other complications. Patient satisfaction was similar between the two groups, but bronchoscopist satisfaction was higher with the DK combination (*p* = 0.033).

**Conclusion:**

DK demonstrated a good safety profile in patients subjected to FB and achieved more profound sedation and better bronchoscopist satisfaction than the standard MF combination without increasing the rate of adverse events.

## Background

Flexible bronchoscopy (FB) is widely used for diagnostic and therapeutic lung interventions. Sedation is routinely administered during the procedure to reduce patient discomfort and facilitate the achievement of the clinical objectives [1]. Since FB is a typically brief process, the ideal agent should ensure deep sedation with preservation of the respiratory drive and minimal side effects [2]. Conventional medications include benzodiazepines (e.g., midazolam), opioids (e.g., fentanyl), and propofol, but all have the disadvantage of potentially significant suppression of the respiratory center [2–5]. Some experts have recently suggested that the evidence supporting the use of the midazolam-fentanyl (MF) combination during FB is weak to moderate [3, 5], while others have even advised against the use of opioids [6].

Dexmedetomidine is a selective α-2 receptor agonist with mild sedative, anxiolytic, and antisialagogue properties [7]. The agent does not affect the respiratory drive but can cause significant hemodynamic instability, mainly bradycardia and hypotension [7, 8]. Several studies have explored the role of dexmedetomidine in FB [9–19]; some of these trials reported a reduced rate of desaturation events compared to the conventional sedatives [9, 11, 13], while others found a higher incidence of hemodynamic adverse events and less satisfactory patient and bronchoscopist experiences [9, 12, 14, 16, 17].

Ketamine is an N-methyl D-aspartate receptor antagonist with anesthetic and powerful analgesic properties [20]. Its adverse events include excessive salivation and agitation on recovery, but the agent offers additional benefits, such as bronchodilation and cardiovascular stimulation (tachycardia, increased blood pressure, increased cardiac output) by enhancing the release of catecholamines [20]. When dexmedetomidine and ketamine are combined, their unfavorable effects may be counterbalanced, resulting in satisfactory sedation with favorable respiratory and hemodynamic profiles [21, 22]. The dexmedetomidine-ketamine (DK) combination has been studied in children [21, 23, 24] and adults requiring sedation for various invasive procedures [19, 22, 25]. The DK combination has also been evaluated as an adjunct to MF [19] and, recently, as a stand-alone regimen [26] in individuals undergoing FB.

The aim of this study was to evaluate further the DK and the standard MF combination in adults undergoing FB in terms of sedation quality, respiratory and hemodynamic profiles, and patient and bronchoscopist satisfaction. We hypothesized that the DK combination would be at least non-inferior to the MF regimen regarding major desaturation events.

## Methods

### Study design and population

This randomized controlled trial of adult patients (age > 18 years) scheduled for FB was conducted between September 2019 and May 2020 at the Respiratory Medicine Department of Athens Naval Hospital, Greece.

Exclusion criteria included: known or suspected allergy to any of the study drugs, renal impairment (serum creatinine > 2 mg/dL), hepatic impairment (liver enzymes > 2 times the upper limit of normal), seizure disorders, history of psychosis or bipolar disorder, hemodynamic instability (heart rate - HR < 50 bpm or systolic blood pressure - SBP < 90 mmHg), and critically ill patients. The study protocol was approved by the Ethics Committee of Athens Naval Hospital (act number 296/13.08.2019), and written informed consent was obtained from all participants.

### Protocol

Demographic data and medical history were reviewed at the presentation, and the vital signs (including the modified Medical Research Council Dyspnea Scale) were assessed.

Fiberoptic and EBUS bronchoscopes were used depending on the target planning of the procedure. EBUS can produce greater discomfort to the patient and can be more demanding for the proceduralist, so an adjusted model was used to score bronchoscopist satisfaction with the different sedation methods.

Eligible patients were then randomly assigned to two groups:

1. *DK group*: Dexmedetomidine solution of 1 µg/kg dissolved in 60 ml saline was administered over 15 min before the procedure was initiated, followed by a maintenance dose of 0.5 µg/kg/h (continuous infusion). After the first 15 min, a bolus dose of 50 mg ketamine dissolved in 10 ml saline was given. A bolus dose of 0.5 µg/kg after at least 20 min as an one-time administration and an increase of 0.1 µg/kg/h in the infusion rate of dexmedetomidine was considered when necessary to optimize the level of sedation.
2. *MF group*: Midazolam was dissolved at the ratio of 1 mg/2 ml saline. We used a premedication dosage of 2 mg midazolam for anxiolysis 15 min before administering the induction dose, while registering vitals and preparing for bronchoscope insertion. Next, an induction dose of 5 mg bolus midazolam was administered and after 3–5 min the bronchoscope was inserted. Bolus doses of 1 mg midazolam at a minimum of 5–7 min intervals were administered as needed in order to obtain the desired sedation depth. Administration of fentanyl was given at a solution of 100 µg fentanyl / 10 ml saline; administered at 50 µg doses. First dose was administered at the induction phase, and up to 2 more bolus doses administered at 20-minute intervals in between and titrated as needed to obtain the desired sedation depth.

The depth of sedation was quantified at the onset of drug infusion using the Observer’s Assessment of Alertness/Sedation scale; a maximum score of 3 was considered optimal for starting FB [27]. Bolus doses of dexmedetomidine (DK group) and midazolam (MF group) were administered when necessary to maintain the same target score. Riker and Ramsay scale scores were also measured for the purposes of comparing sedation scales in regards to outcomes as a possible secondary endpoint. An anesthesiologist was present throughout the procedure to oversee and monitor the sedation protocol. Only the anesthesiologist and the nurse of the bronchoscopy suite were aware of the sedation regimen. The attending pulmonologist remained blind regarding the sedation protocol until the completion of the bronchoscopist satisfaction questionnaire (see below).

Lidocaine gel was placed at the nostril of entry before the procedure, and lidocaine (4 ml of 2% solution) was sprayed under direct vision through the bronchoscope for vocal cord anesthesia before entering the larynx. All participants were initially premedicated with 2 mg of midazolam 20 min before commencing the procedure for anxiolysis and perioperative amnesia. They also received oxygen at 2 L/min via nasal cannula and 0.9%.

NaCl i.v. at 8 ml/h throughout the procedure. Continuous monitoring included electrocardiography, oxygen saturation (SpO_2_), and automated non-invasive blood pressure recordings.

### Outcomes

The total dose of the administered sedatives (including boluses) was recorded for each patient. The duration of the procedure (duration of FB) and recovery (measured from the time of bronchoscope withdrawal until the time the patient was evaluated as ready for discharge) were also noted.

The depth of sedation was evaluated using the Ramsay sedation scale [28] and the Riker sedation-agitation scale [29]. Both tools provide quantitative measurements in the maximum and minimum sedation-agitation range during the procedure. Higher Ramsay and lower Riker scores signify deeper sedation levels [28, 29].

Cough rate and intensity were evaluated according to a previously published questionnaire [30] including Likert-scale [36] items, as follows: 1 = no cough before or during the procedure; 2 = cough before FB without interruption of the procedure; 3 = cough during the procedure which demands more than one interruptions; 4 = persistent cough with more than three (frequent) procedural interruptions; 5 = persistent cough with frequent procedural interruptions and residual cough for more than two hours post-procedure. The rates of adverse events, including desaturations (SpO_2_ < 80%), blood pressure instability (determined by having a > 20% fluctuation of pre-operative mean arterial pressure) and significant bleeding (determined as bleeding on bronchoscopy site needing chemical intervention, wedging, mechanical ventilation or surgical intervention) were recorded.

Bronchoscopist satisfaction was measured with a Likert-scale tool answering the question *“How satisfied are you with both the ease and outcome of the procedure”* as follows: 1 = not satisfied at all; 2 = somewhat satisfied; 3 = more satisfied with the procedural outcome; 4 = mostly satisfied with the outcome and somewhat with procedural ease; 5 = exceptionally satisfied with both procedural outcome and ease.

Patient satisfaction [31] was evaluated by the following questions: (1) I was satisfied with the sedation administered for the procedure, (2) I felt pain and/or discomfort beyond my tolerance during the procedure, (3) I believe my needs were met during the procedure (4) I felt pain and discomfort after the procedure, (5) I would be willing to undergo a second procedure if the first did not yield adequate results. The responses were measured in a Likert scale format with values 1 = strongly disagree; 2 = disagree; 3 = neither agree nor disagree; 4 = agree; 5 = strongly agree. The questionnaire was given in printed form at discharge, and the responses were collected by telephone call the day after the procedure.

### Sample size estimation and statistical analyses

The primary endpoint of the study was the occurrence of major desaturation events (i.e., SpO_2_ < 80% with nasal oxygen supply 2 L/min, duration >/= 15 s). Assuming a desaturation rate of 20 ± 5%, we calculated a lower critical number of 25 patients per group to prove non-inferiority within the above limits of the primary endpoint, with a *p*-value of < 0.05 and 85% power.

Continuous variables are presented as mean ± SD with median and range and compared with the Student’s t or Mann-Whitney U test, as appropriate. Categorical variables are given as number of cases (%) and compared with the chi-square or Fisher’s exact test. Multivariable linear regression analysis was applied to reveal the predictors of bronchoscopist satisfaction (log-transformed score). A *p*-value of < 0.05 was considered significant in all instances. Statistical analysis was conducted with the SPSS version 28 (IBM Corp., Armonk, NY).

## Results

**Fig. 1:**
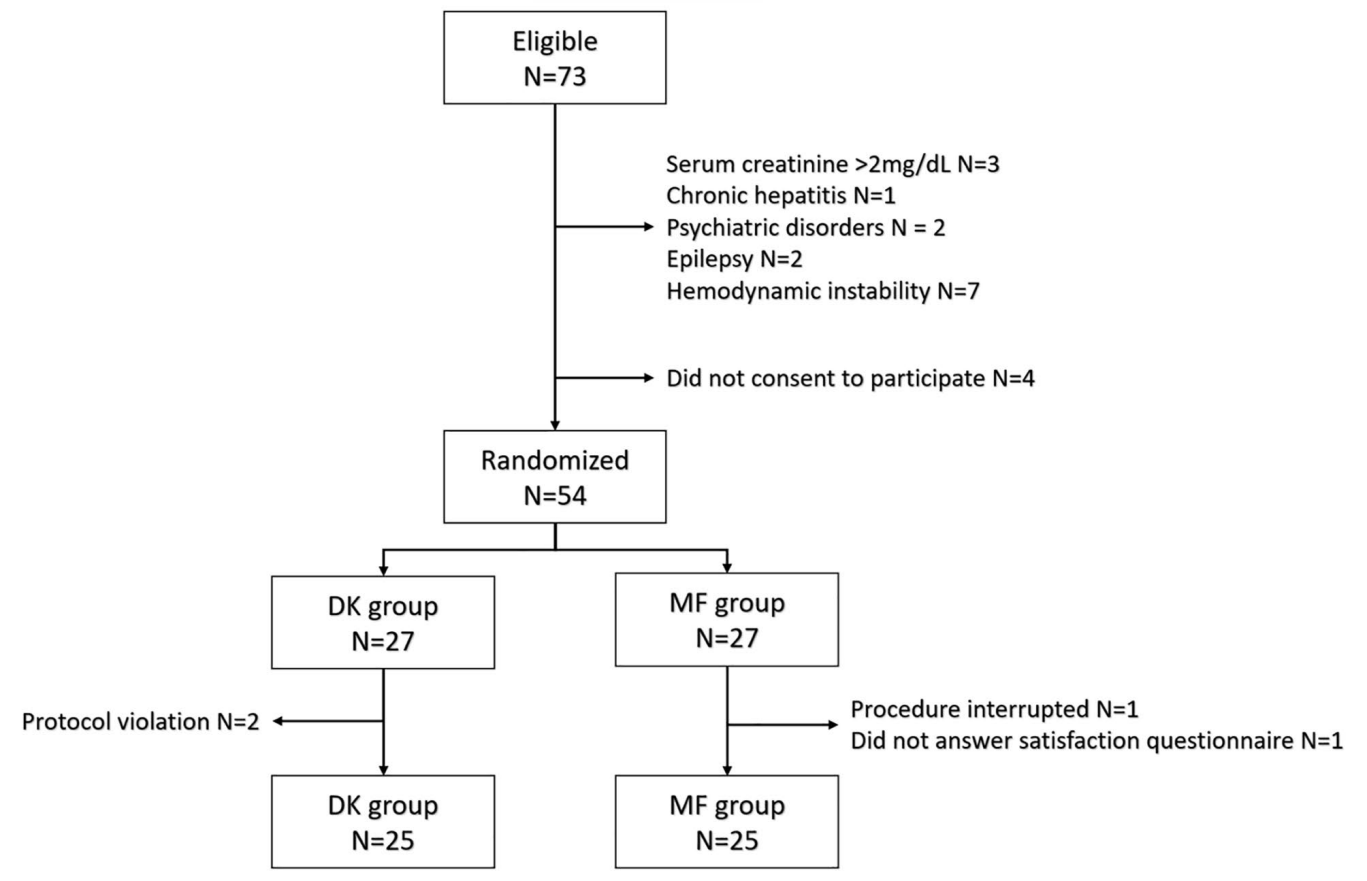
Study flow. MF: Midazolam - Fentanyl, DK: Dexmedetomidine - Ketamine

A total of 73 patients were screened for eligibility during the study period. Of them, 15 did not fulfill the inclusion criteria, and 4 did not consent to participate in the study. The remaining 54 patients were randomly allocated to the two study groups. The DK group noted two sedation protocol violations (the attending anesthesiologist changed to a different drug). In the MF group, the procedure was interrupted in one patient due to severe bleeding, while one participant refused to answer the patient satisfaction questionnaire. Therefore, the final study population included 50 patients, 25 in each group. Their demographic and clinical characteristics are shown in Table 1. A study flow is presented in Fig. 1.

Indications for bronchoscopy for the MF and DK group included: pulmonary nodule 5 (20%) and 15 (60%), mass 9 (36%) and 5 (20%), consolidation 9 (36%) and 6 (24%), and lymph node enlargement 3 (12%) and 0 (0%), respectively. A simple fiberoptic bronchoscope was used in 19 (76%) of the MF group patients and in 14 (56%) of those of the DK group (*p* = 0.232). An endobronchial ultrasound (EBUS) bronchoscope was used in 6 (24%) patients of the MF group and 11 (44%) of the DK group.

**Fig. 2:**
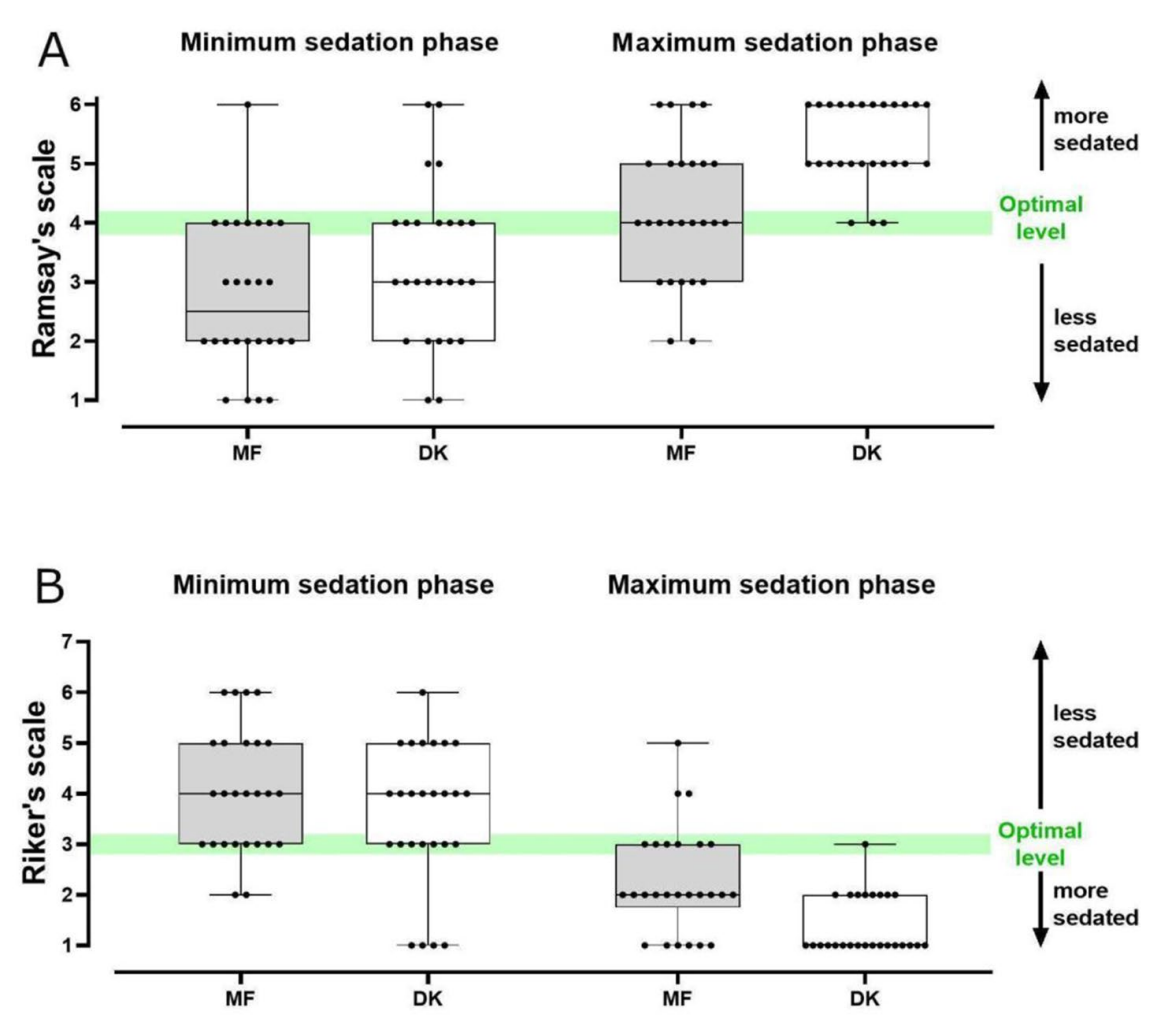
Sedation levels of study participants per sedation phase and protocol. The box-whisker plots demonstrate the Ramsay and Riker scale for estimating sedation level. MF: Midazolam - Fentanyl, DK: Dexmedetomidine - Ketamine

**Fig. 3:**
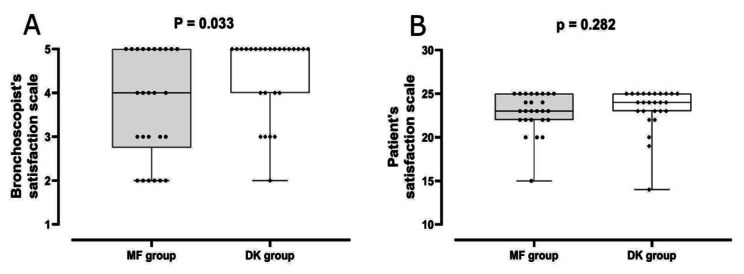
Bronchoscopist and patient satisfaction levels. MF: Midazolam - Fentanyl, DK: Dexmedetomidine - Ketamine. Abrevations: FB: Fiberoptic Bronchoscopy; MF: Midazolam and Fentanyl; DK: Dexmedetomidine and Ketamine; HR: Heart Rate; SBP: Systolic Blood Pressure; EBUS: Endobronchial Ultrasound

The study outcomes are presented in Table 2 and in Figs. 2 and 3. The DK combination achieved deeper sedation levels at the phase of maximum sedation compared to the MF regimen (Table 2). During that phase, most patients in the DK group were well beyond the optimal sedation level (i.e., they were more sedated) (Fig. 2). Sedation characteristics during the minimum sedation phase were comparable between the two groups (Table 2; Fig. 2). DK combination was associated with longer procedure and recovery duration (Table 2). The DK group scored marginally lower on the cough scale (*p* = 0.064). The rate of critical desaturation events was 28% in the MF and 12% in the DK group (*p* = 0.289). The rate of other complications also did not differ (Table 2). One female patient in the DK group presented urinary loss during the procedure without prior medical history of incontinence.

Bronchoscopist satisfaction was higher in the DK group (4.4 ± 0.9; median 5, range 2–5) compared to the MF group (3.7 ± 1.2; median 4, range 2–5; *p* = 0.033) (Fig. 3). The DK combination and the lack of complications during the procedure emerged as significant and independent determinants of bronchoscopist satisfaction (Table 3). Multivariable regression analysis showed a statistically significant value of *p* = 0.018 for the DK combination adjusted effect, while a value of *p* = 0.596 for EBUS usage in regards to bronchoscopist satisfaction, which suggests that EBUS usage did not affect bronchoscopist satisfaction. Patient satisfaction levels were comparable between the DK (23.3 ± 2.5; median 24, range 14–25) and the MF group (22.9 ± 2.3; median 23, range 15–25; *P* = 0.282) (Fig. 3).

## Discussion

In this single-center, randomized controlled trial, we explored the safety and efficacy of the DK combination compared to a standard MF regimen in adults undergoing FB. Our findings suggest that DK can achieve deep sedation with adequate respiratory and hemodynamic profiles, reasonable patient comfort, and high bronchoscopist satisfaction. Major desaturation events occurred at a similar frequency in the two study groups, thus confirming the non-inferiority of the DK combination, in line with the study hypothesis. However, the time required for recovery was significantly longer in the DK group, which might limit the widespread application of the DK combination, especially in the case of busy bronchoscopic laboratories.

Dexmedetomidine, as opposed to conventional sedatives and opioids, has the advantage of preserving the respiratory drive, a critical objective of conscious sedation during bronchoscopy [1, 2]. However, the drug may cause hemodynamic instability (i.e., bradycardia and hypotension), which may significantly affect the success of the interventional procedure [8]. Indeed, previous studies in adults have confirmed that dexmedetomidine was associated with more hemodynamic adverse events leading to less satisfactory doctor experiences [9, 12, 14, 16, 17]. Ketamine, on the other hand, despite its well-known adverse effects, causes cardiovascular stimulation, which may counterbalance the effects of dexmedetomidine [21]. In addition, ketamine is a potent analgesic that could act complementary to dexmedetomidine to increase patient comfort [21, 22]; its bronchodilatory effect and the ability to preserve the respiratory drive at low doses [20] may offer additional benefits in patients undergoing FB [1].

An earlier randomized-controlled trial has explored the utility of DK combination as an adjunct to MF regimen in adults undergoing FB [19]. Atkins et al. [19] have shown that, patients in the MF-DK group (*n* = 25) achieved lower serum midazolam and fentanyl levels than those (*n* = 25) who received only the MF combination. Patient and bronchoscopist satisfaction scores and time of desaturations (SpO_2_ < 90%) were comparable between the two groups. It is worth noting that the relative decrease in minute ventilation was greater in the MF group [19]. Although the design and the objectives of the above study were different from ours (DK as adjuvant therapy [19] versus DK as a stand-alone regimen in our study), its findings are in line with ours; in patients undergoing FB, since the DK combination can achieve satisfactory conscious sedation without significant respiratory or hemodynamic adverse events.

The DK combination has also been studied in children [21, 23, 24] and adults [22, 25] undergoing invasive procedures other than FB. Similar to our study, those trials have proven the safety and efficacy of the DK combination in terms of sedation quality, respiratory and hemodynamic complications, and patient and doctor satisfaction. However, the time required to obtain the appropriate level of sedation and the duration of recovery was generally longer than those observed with the conventional regimens. Thus, it has been recently suggested that the DK combination may be more suitable for high-risk patients who require hemodynamic and respiratory stability during non-urgent invasive procedures [25].

Airway interventions like bronchoscopy have specific requirements related to cough, gag reflex, and patient agitation due to discomfort [1]. In this regard, the DK combination performed optimally in our study, which may explain the higher bronchoscopist satisfaction in the DK group. The deeper sedation level most likely facilitated the attending bronchoscopist and allowed for a more focused and thorough procedure; Additionally, the significantly longer duration of FB in the DK group further supports the above hypothesis. Future studies should explore this aspect by taking into account the qualitative characteristics of bronchoscopist and anesthesiologist satisfaction in regards to the intervention type (FB, EBUS, etc.) and the particularities of each patient, as a personalized approach.

Major desaturation events occurred with statistically similar frequencies in the two study groups, albeit their rate was slightly lower with the DK combination. Although this result supports the non-inferiority of the DK regimen, it does not indicate that DK may be more suitable for high-risk respiratory patients [21]. A recent study that investigated the role of dexmedetomidine in awake fiberoptic intubation for the management of difficult airway reported a markedly decreased muscle tone compared to fentanyl and ketamine [32], suggesting that this may be the principal mechanism of dexmedetomidine-induced respiratory depression. This is a different mechanism to the central respiratory depression caused by benzodiazepines and opiates. Reduced muscle tone respiratory depression can be less impactful to certain patient populations like Parkinson’s disease patients, and can be usually dealt with a jaw thrust maneuver, which we successfully used in our cases as well. Riker scale score and Ramsay scale measurement analysis did not offer any additional insight, but the data might prove to be useful for a cross-examination of different scales for procedural sedation in regards to patient and bronchoscopist satisfaction in future meta-analysis.

Notably, the DK combination might prove more suitable for patients with Parkinson’s disease, in whom respiratory-related disease incidences are pervasive [33]. While benzodiazepines can aggravate Parkinson’s symptoms [34], dexmedetomidine and ketamine have been shown to ameliorate dyskinesia and improve the respiratory mechanics in such patients [35, 36].

Our study has limitations. The sample size, although proper to prove non-inferiority, was small and did not offer enough power to evaluate further the apparent benefits of the DK regimen (e.g., in terms of cough and major desaturation events) or to detect uncommon complications in either treatment arm. Second, our study was only partially blinded; although the pulmonologist was unaware of the sedation protocol, the attending anesthesiologist and the laboratory nurse had access to the type of sedation. Third, the study sample was rather inhomogeneous, including patients with various respiratory diseases and different procedural goals. Fourth, we have no data on the exact number of midazolam/fentanyl bolus doses used in the MF group, and no data for the patients for whom an increase in the infusion rate of dexmedetomidine was required in the DK group. Finally, it is possible that the midazolam premedication offered a synergistic action with dexmedetomidine and ketamine. Nevertheless, the sedation protocol remained consistent across all participants. Combining DK with an anxiolytic dose of midazolam may prove even more effective for demanding bronchoscopic procedures, omitting the use of fentanyl to maximize sedation while minimizing impact on respiratory function. Future studies, with better design and larger sample sizes, should also determine the appropriate dosage of DK combination and explore its benefits over the conventional sedation regimens for specific patient populations (e.g., high-risk respiratory patients, patients with Parkinson’s disease, patients on opiate withdrawal or with contraindications to opiates, etc.).

In conclusion, our study showed that the administration of a DK combination during FB achieved conscious sedation, stable respiratory and hemodynamic profiles, and patient comfort comparable to those of the classical MF regimen. Although the time required for recovery was longer in the DK group, the deeper but uncomplicated sedation allowed for a more focused and thorough procedure, thus resulting in significantly higher bronchoscopist satisfaction. The DK combination may be considered a viable and promising alternative to standard sedation regimens in adult patients undergoing FB.

**Table 1:** Characteristics of the study groups

	MF	DK	*p*-value
N	25	25	
Male sex	19 (76)	16 (64)	0.537
Age, years	67.5 ± 11.7 (68; 38–86)	62.7 ± 15 (68; 28–80)	0.415
Height, cm	172 ± 7.7 (175; 150–185)	171.2 ± 8 (170; 155–185)	0.495
Weight, kg	78.8 ± 10.2 (79; 60–100)	81.9 ± 19.2 (84; 50–128)	0.424
Heart rate, min^–1^	74 ± 14 (73; 45–100)	71 ± 12.8 (70; 50–99)	0.470
Respiratory rate, min^–1^	14.1 ± 3.3 (14; 10–25)	15.2 ± 4.6 (14; 9–25)	0.506
Systolic blood pressure, mmHg	136.5 ± 16.8 (135; 110–165)	136.2 ± 16.2 (135; 110–160)	0.956
Diastolic blood pressure, mmHg	75.8 ± 11.4 (80; 50–90)	73.8 ± 9.4 (80; 60–90)	0.498
Oxygen saturation, %	96.9 ± 2.3 (98; 93–99)	97.7 ± 1.4 (98; 93–99)	0.438
Dyspnea scale*	1.4 ± 0.9 (1; 1–4)	1.3 ± 0.5 (1; 1–3)	0.862

Data are presented as mean ± SD (median; range) or number of cases (%) and compared with Mann-Whitney U or chi-square test, as appropriate.

* modified Medical Research Council Dyspnea Scale

NF: Midazolam - Fentanyl, DK: Dexmedetomidine - Ketamine

**Table 2:** Outcomes and adverse events

	MF	DK	*p*-value
Duration of procedure, min	40 ± 11.7 (40; 30–80)	47.7 ± 13.9 (42.5; 30–90)	0.016
Ramsay’s sedation scale			
Maximum	4.2 ± 1.2 (4; 2–6)	5.4 ± 0.7 (5; 4–6)	< 0.001
Minimum	2.7 ± 1.3 (2.5; 1–6)	3.3 ± 1.3 (3; 1–6)	0.114
Difference max- min	1.5 ± 1.6 (1; 2–5)	2 ± 1.6 (2; 2–5)	0.154
Riker’s sedation-agitation scale			
Maximum	4 ± 1.2 (4; 2–6)	3.6 ± 1.4 (4; 2–6)	0.375
Minimum	2.3 ± 1 (2; 1–5)	1.4 ± 0.6 (2; 1–3)	< 0.001
Difference max-min	1.8 ± 1.7 (1; 2–5)	2.2 ± 1.7 (2; 1–5)	0.306
Cough scale	2.3 ± 1.2 (2; 1–5)	1.8 ± 1.1 (1; 1–5)	0.064
Complications			
Desaturation, n (%)	7 (28)	3 (12)	0.289
Hypertension, n (%)	8 (32)	5 (20)	0.519
Hypotension, n (%)	2 (8)	4 (16)	0.663
Bradycardia, n (%)	3 (12)	1 (4)	0.602
Bleeding, n (%)	3 (12)	2 (8)	0.999
Duration of recovery, min	29.6 ± 12.5 (31; 10–52)	54.6 ± 33.9 (55; 10–120)	< 0.001

Data are presented as mean ± SD (median; range) or number of cases (%) and compared with Mann-Whitney U or chi-square/Fisher’s exact test, as appropriate.

NF: Midazolam - Fentanyl, DK: Dexmedetomidine - Ketamine

**Table 3:** Predictors of bronchoscopist satisfaction

	Unadjusted effect	Adjusted effect
DK combination	0.257 (0.065)	0.329 (0.018)
EBUS bronchoscope	0.042 (0.769)	0.071 (0.596)
No complications	0.309 (0.026)	0.351 (0.010)

Data are linear regression coefficients with *p*-values in parentheses. The log-transformed satisfaction score was used as the dependent variable.

The unadjusted effect refers to the effect of each variable separately. The adjusted effect refers to the effect of these parameters adjusted for each other (multivariable regression analysis).

DK: Dexmedetomidine - Ketamine

## Data Availability

The datasets used and/or analyzed during the current study available from the corresponding author on reasonable request.
